# Supplementary material for: Outcome Analysis of the Use of Cerament® in Patients with Chronic Osteomyelitis and Corticomedullary Defects
Source: Diagnostics (Basel). 2022 May 11;12(5):1207. doi: 10.3390/diagnostics12051207 (PMC9139791; doi:10.3390/diagnostics12051207)
Supplement: Supplementary file 1 [file diagnostics-12-01207-s001.zip › diagnostics-1682211-supplementary.pdf]

Supplementary Table S1. In-depth characteristics of the study cohort.

Patient number	Gender	Age (years)	BMI	CCI	ASA	Previous revision surgeries	Disease duration (months)	OM location	OM stage	Size of defect (cm^3)	Defect filler	LOS (days)	Intravenous antibiotics (days)	Oral antibiotics (days)	Following revisions	Duration until revision (days)	Follow up (months)
1	male	33	41	0	1	n. a.	28	Tibia	III A	8.8	Cerament® G	20	26	164	2	182	33
2	female	41	25	2	2	17	236	Calcaneum	III B^{s}	8.8	Cerament® G	16	16	11	3	26	6
3	male	39	24	0	1	4	12	Humerus	III A	12.9	Cerament® V	28	7	0	2	6	3
4	male	54	23	2	2	3	8	Humerus	III B^{l}	14.5	Cerament® G	14	12	33	0	n. a.	24
5	male	73	24	2	3	n. a.	490	Tibia	III B^{s}	0.1	Cerament® V	15	11	0	1	91	4
6	male	59	26	1	1	2	526	Tibia	III A	8.4	Cerament® G	17	21	0	0	n. a.	31
7	male	54	21	7	2	3	29	Femur	III B^{ls}	4.3	Cerament® G	14	13	30	0	n. a.	4
8	female	48	19	0	2	60	327	Femur	III A	6.0	Cerament® G	63	36	0	0	n. a.	46
9	female	19	19	2	2	0	27	Tibia	III B^{s}	23.1	Cerament® G	8	15	7	1	39	59
10	male	26	24	0	1	6	74	Tibia	III A	4.6	Cerament® G	21	5	1	0	n. a.	48
11	female	46	28	1	2	6	72	Tibia	III B^{l}	5.1	Cerament® G	14	15	29	0	n. a.	5
12	male	45	30	0	2	1	93	Tibia	III B^{l}	3.3	Cerament® G	33	13	24	2	19	17
13	female	73	20	2	2	0	11	Calcaneum	III B^{s}	0.9	Cerament® G	14	12	42	n. a.	n. a.	40
14	female	70	26	4	3	2	44	Ulna	III B^{ls}	1.4	Cerament® G	25	64	14	0	n. a.	5
15	male	43	20	0	2	n. a.	344	Femur	III B^{s}	4.6	Cerament® V	38	17	70	2	24	18
16	male	64	28	2	2	3	69	Tibia	III B^{s}	2.9	Cerament® V	15	15	72	0	n. a.	18
17	male	28	27	0	2	3	137	Tibia	III A	1.7	Cerament® V	15	12	32	0	n. a.	9
18	female	23	20	0	1	0	78	Tibia	III A	2.0	Cerament® G	7	15	29	0	n. a.	4
19	female	36	25	0	1	n. a.	22	Calcaneum	III A	8.8	Cerament® G	14	45	0	1	150	9
20	male	54	23	1	2	30	366	Tibia	III B^{s}	1.1	Cerament® G	28	8	36	5	4	21

Abbreviations: BMI: Body mass index, CCI: Charlson comorbidity index, ASA: American Society of Anaesthesiologists' physical status classification system, OM: Osteomyelitis, III A: Localized osteomyelitis in a normal host, III B^{L}: Localized osteomyelitis in a locally compromised host, III B^{S}: Localized osteomyelitis in a systemically compromised host, III B^{LS}: Localized osteomyelitis in a locally and systemically compromised host, LOS: Length of stay, n. a.: Not available due to missing data.

Supplementary Table S2. Clinical outcome measures of the study cohort.

		N (n revised/ n not revised)	Total sample (N = 10)	Revised (N = 6)	Not revised (N = 4)	Statistic*
DASH	Global	2 (1/1)	28.4 ± 8.3 (95 % CI −46.0–102.7)	34.2	22.5	n. a.
	Sport module	2 (1/1)	59.4 ± 22.1 (95 % CI −139.2–257.9)	43.8	75.0	n. a.
	Work module	2 (1/1)	9.4 ± 13.3 (95 % CI −109.7–128.5)	18.8	0.0	n. a.
KSS	Objective	3 (1/2)	55.0 ± 8.9 (95 % CI 32.9–77.1)	58.0	53.5 ± 12.0 (95 % CI −54.5–161.5)	n. a.
	Satisfaction	3 (1/2)	27.3 ± 11.4 (95 % CI −0.9–55.6)	40.0	21.0 ± 4.2 (95 % CI −17.1–59.1)	n. a.
	Expectation	3 (1/2)	8.0 ± 1.7 (95 % CI 3.7–12.3)	9.0	7.5 ± 2.1 (95 % CI −11.6–26.6)	n. a.
	Functions	3 (1/2)	75.7 ± 4.6 (95 % CI 64.2–87.1)	81.0	73.0 ± 0.0 (95 % CI 73.0–73.0)	n. a.
FAOS	Total	5 (4/1)	49.4 ± 26.7 (95 % CI 16.3–82.6)	44.3 ± 27.8 (95 % CI 0.0–88.5)	70.0	n. a.
	Symptoms & stiffness	5 (4/1)	53.0 ± 27.4 (95 % CI 19.0–87.0)	49.3 ± 30.1 (95 % CI 1.4–97.1)	68.0	n. a.
	Pain	5 (4/1)	64.4 ± 38.6 (95 % CI 16.5–112.3)	59.0 ± 42.3 (95 % CI −8.4–126.4)	86.0	n. a.
	ADL	5 (4/1)	56.0 ± 32.8 (95 % CI 15.2–96.8)	49.8 ± 34.3 (95 % CI −4.9–104.4)	81.0	n. a.
	Sport	5 (4/1)	7.0 ± 13.0 (95 % CI −9.2–23.2)	1.3 ± 2.5 (95 % CI −2.7–5.2)	30.0	n. a.
	QoL	5 (4/1)	35.0 ± 38.8 (95 % CI −13.2–83.2)	34.3 ± 44.7 (95 % CI −36.9–105.4)	38.0	n. a.
SF-36	Physical functioning	10 (6/4)	44.0 ± 32.4 (95 % CI 20.8–67.2)	30.0 ± 26.8 (95 % CI 1.8–58.2)	65.0 ± 31.1 (95 % CI 15.5–114.5)	$p = 0.1$
	Physical health	10 (6/4)	37.5 ± 41.3 (95 % CI 8.0–67.0)	25.0 ± 41.8 (95 % CI −18.9–68.9)	56.3 ± 37.5 (95 % CI −3.4–115.9)	$p = 0.2$
	Emotional problems	10 (6/4)	66.7 ± 41.6 (95 % CI 36.9–96.4)	55.6 ± 45.6 (95 % CI 7.8–103.4)	83.3 ± 33.4 (95 % CI 30.3–136.4)	$p = 0.4$
	Energy/fatigue	10 (6/4)	54.0 ± 23.6 (95 % CI 37.2–70.8)	45.0 ± 25.1 (95 % CI 18.7–71.3)	67.5 ± 14.4 (95 % CI 44.5–90.5)	$p = 0.2$
	Emotional well-being	10 (6/4)	68.0 ± 21.6 (95 % CI 52.6–83.4)	60.0 ± 25.0 (95 % CI 33.7–86.3)	80.0 ± 5.7 (95 % CI 71.0–89.0)	$p = 0.2$
	Social functioning	10 (6/4)	67.5 ± 32.9 (95 % CI 44.0–91.1)	54.2 ± 32.3 (95 % CI 20.3–88.0)	87.5 ± 25.0 (95 % CI 47.7–127.3)	$p = 0.2$
	Pain	10 (6/4)	59.5 ± 28.4 (95 % CI 39.2–79.8)	57.9 ± 36.4 (95 % CI 19.7–96.1)	61.9 ± 13.9 (95 % CI 39.8–84.0)	$p = 1$
	General health	10 (6/4)	52.0 ± 17.4 (95 % CI 39.6–64.4)	52.5 ± 16.1 (95 % CI 35.7–69.3)	51.3 ± 21.8 (95 % CI 16.7–85.9)	$p = 0.9$
	NRS	10 (6/4)	3.2 ± 3.1 (95 % CI 1.0-5.4)	3.5 ± 3.8 (95 % CI −0.5–7.5)	2.8 ± 1.9 (95 % CI −0.3–5.8)	$p > 0.9$

*Statistical analysis comparing patients with and without revision.

Abbreviations: DASH: Disabilities of the Arm, Shoulder, and Hand Score, KSS: Knee Society Score, FAOS: Foot and Ankle Outcome Score, SF-36: Short-form Health Survey 36-item Score, NRS: Numeric Rating Scale, n. a.: not available due to insufficient number of cases
